# 

**Published:** 1906-03

**Authors:** 


### CONTENTS.

ORIGINAL COMMUNICATIONS—

Prostatic Albuminuria not an Infrequent Cause of Error in the Diagnosis of the So-called "Orthostatic," "Postural," "Physiological" and "Cyclic" Albuminueia. By Edgar G. Ballenger, M.D., Atlanta, Ga. .......... 793

The New Pharmacopœia and the Proprietaries. By Samuel A. Visanska, Ph.G., M.D, Atlanta, Ga. .......... 800

Morphine and Scopolamin as a Preliminary to Anesthesia. By K. Lowder Reid, M.D., Atlanta Ga. .......... 804

The After-Care of Obstetrical Patients. By Stephen T. Barnett, M.D., Atlanta, Ga. .......... 807

Conditions Found in Chronic Rheumatism of the Knees. By Michael Hoke, M.D., Atlanta, Ga. .......... 819

EDITORIAL NOTES AND COMMENTS—

In or Out?. .......... 829

The Journal-Record the Official Organ of the Fulton County Medical Society. .......... 830

NEWS AND NOTES. .......... 831

CONTENTS CONTINUED. .......... x

